# H19 and the PD-1/PD-L1 Axis in hepatocellular carcinoma and colorectal cancer: emerging evidence, mechanistic links, and therapeutic perspectives

**DOI:** 10.1007/s10238-026-02319-9

**Published:** 2026-09-06

**Authors:** Mohammad Taha Nami, Fatemeh EmamiPari, Nooshin Farrokhi, Iliya Fattahyan, Maryam Yekkalam

**Affiliations:** 1 Department of Cell and Molecular Biology, Faculty of Life Science and Biotechnology, Shahid Beheshti University, Tehran, Iran; 2 Clinical Research Development Center, Amir Oncology Teaching Hospital, Shiraz University of Medical Sciences, Shiraz, Iran

**Keywords:** Hepatocellular carcinoma, Colorectal cancer, LncRNA H19, PD-1/PD-L1, Immune evasion, Tumor microenvironment

## Abstract

Hepatocellular carcinoma (HCC) and colorectal cancer (CRC) are major causes of cancer-related mortality, yet responses to immune checkpoint inhibitors (ICIs) remain heterogeneous. Although programmed death-ligand 1 (PD-L1) is a key component of immune checkpoint biology, antitumor immunity is shaped by multiple tumor-intrinsic and microenvironmental factors, and the contribution of the long non-coding RNA H19 to these processes remains incompletely defined. This narrative review synthesizes experimental and clinical evidence linking H19 to immune regulation in HCC and CRC, with particular emphasis on its potential intersection with PD-L1-mediated immune checkpoint signaling. Evidence was classified as experimentally demonstrated, indirectly supported, contextual, or hypothetical according to the strength, mechanistic resolution, and disease specificity of the available data. Current evidence indicates that H19 perturbation can influence PD-L1-associated phenotypes in selected experimental HCC models; however, a complete, mechanistically resolved H19-dependent PD-L1 regulatory axis, including validated intermediate mechanisms and causal rescue, has not been established in HCC or CRC. Proposed ceRNA and signaling mechanisms remain incompletely validated, while several mechanistic links are supported primarily by indirect or cross-cancer evidence. Beyond direct checkpoint regulation, H19-associated extracellular vesicles and H19-dependent signaling have been implicated in macrophage remodeling, intercellular communication, and T-cell dysfunction, although direct convergence of these processes on H19-mediated PD-L1 regulation remains unproven. Circulating H19 has also shown preliminary associations with tumor presence or burden, but its clinical utility as a diagnostic, prognostic, or predictive biomarker remains insufficiently validated, particularly for ICI response. Overall, H19 represents a biologically plausible interface between non-coding RNA regulation, tumor-intrinsic signaling, and the tumor immune microenvironment rather than an established central regulator of PD-L1. Disease-specific mechanistic studies incorporating H19 perturbation, PD-L1 measurement, pathway-specific rescue, and immune-functional assays are required to determine whether H19 can serve as a clinically actionable component of immune checkpoint regulation.

## Introduction

Hepatocellular carcinoma (HCC) is a devastating primary liver cancer that mostly develops in patients with chronic liver diseases and cirrhosis [1]. Colorectal cancer (CRC), another major global health burden, arises in the colon or rectum and commonly develops from precancerous polyps that may progress to malignancy over time [2]. In 2022, liver cancer was responsible for almost 865,000 new cases and 758,000 deaths globally. It ranks sixth in incidence and third in cancer-related mortalities. Similarly, CRC accounted for nearly 1.93 million new cases and 904,000 death ranking as the third most commonly diagnosed cancer and the second leading cause of cancer-related death [3].

Despite advances in surgery, transplantation, radiotherapy, and systemic treatment, outcomes remain heterogeneous, particularly in advanced disease, and immunotherapy has become an important component of contemporary cancer treatment [4]. Several immunotherapeutic strategies have been developed, including cytokine-based approaches, such as interleukin-2 (IL-2) and interferons (IFNs), chimeric antigen receptor (CAR) T-cell therapy, and, most prominently, immune checkpoint inhibitors (ICIs) targeting pathways such as programmed cell death protein 1 (PD-1)/programmed death-ligand 1 (PD-L1) [5, 6]. However, primary or acquired resistance remains common, response rates vary substantially among tumor types and patients, and immune-related adverse events can limit treatment [7].

The PD-1/PD-L1 axis is a major pathway involved in tumor immune evasion. Engagement of PD-1 by PD-L1 can inhibit T-cell signaling and contribute to T-cell dysfunction and exhaustion [8]. Importantly, the clinical activity of immune checkpoint blockade is not determined by PD-L1 expression alone. Antigenicity, interferon signaling, antigen processing and presentation, immune-cell trafficking and exclusion, stromal architecture, and immunosuppressive myeloid populations can all influence whether an immune-permissive tumor microenvironment is established and whether checkpoint blockade produces durable responses [9]. Thus, PD-1/PD-L1 signaling should be considered one component of a broader and context-dependent network of tumor–immune interactions. CRC provides a clear example of this context dependence: microsatellite instability-high (MSI-H)/mismatch repair-deficient (dMMR) CRC demonstrates substantial sensitivity to PD-1 blockade, whereas microsatellite-stable (MSS) CRC is generally less responsive to single-agent checkpoint inhibition, as illustrated by the KEYNOTE-177 study [69].

Long non-coding RNAs (lncRNAs) are transcripts longer than 200 nucleotides that regulate gene expression through diverse mechanisms, including RNA–RNA interactions, transcriptional regulation, protein scaffolding, and modulation of signaling pathways [10]. Among these, H19 is an imprinted oncofetal lncRNA that is frequently dysregulated in cancer and has been implicated in tumor progression, treatment resistance, cellular plasticity, and tumor–microenvironment communication. H19 can function as a competing endogenous RNA (ceRNA), thereby modulating the availability of specific microRNAs (miRNAs), and may also participate in oncogenic and inflammatory signaling networks and extracellular-vesicle (EV)-mediated intercellular communication [11, 12].

These properties make H19 a biologically plausible candidate for investigation within immune-regulatory networks. However, the available evidence does not establish H19 as a universal or central regulator of PD-L1 in HCC or CRC. In particular, observations involving H19, miRNAs, and PD-L1 should be interpreted as distinct levels of evidence rather than as components of a single experimentally validated pathway. In CRC, H19-associated miRNA observations and independent evidence that miR-200a or miR-138-5p can influence PD-L1-related biology provide potentially relevant mechanistic intersections, but these separate findings do not establish a direct H19/miRNA/PD-L1 axis in CRC [83]. Similarly, in HCC, evidence concerning H19-associated regulation of miR-140-5p and independent studies of PD-L1-regulatory pathways provide biological plausibility for a possible intersection; however, direct experimental evidence connecting H19 to PD-L1 remains limited [15].

Although HCC and CRC are biologically distinct malignancies, they provide complementary contexts in which to examine H19 within tumor–immune regulation. Both develop within complex microenvironments involving inflammatory, stromal, metabolic, cytokine, and myeloid components [13]. Their biological relationship is further informed by the gut–liver axis, through which microbial and immune signals can influence hepatic inflammation and immunity. CRC liver metastasis also provides an additional context in which intestinal tumor biology interacts with the hepatic microenvironment and pre-metastatic niche formation [14]. Examining H19 across HCC and CRC therefore permits comparison of potentially shared mechanisms while preserving disease-specific differences in tumor biology, immune context, and therapeutic response.

The novelty of this review does not rest on the proposition that H19 has already been established as a regulator of PD-L1. Rather, we provide a critical evidence map of H19-related experimental and clinical observations that intersect with immune checkpoint biology in HCC and CRC, while explicitly distinguishing experimentally demonstrated mechanisms from indirect, contextual, and hypothetical relationships.

## Review methodology

This narrative review was conducted to critically evaluate the evidence linking the long non-coding RNA H19 to the PD-1/PD-L1 immune checkpoint axis in hepatocellular carcinoma (HCC) and colorectal cancer (CRC). A structured literature search was performed in PubMed/MEDLINE, Scopus, and Web of Science from database inception to July 2026. The principal search terms and Boolean combinations included: “H19” OR “lncRNA H19” AND (“PD-L1” OR “CD274” OR “PD-1” OR “PDCD1”) AND (“hepatocellular carcinoma” OR “HCC” OR “liver cancer” OR “colorectal cancer” OR “CRC” OR “colon cancer”). Additional terms related to extracellular vesicles, macrophages, microRNAs, immunotherapy, STAT3, PI3K/AKT, and NF-κB were used in secondary searches to capture relevant mechanistic studies. Primary experimental studies (in vitro, in vivo, and human tissue analyses) were prioritized over secondary literature. Review articles were retained only for background orientation and were not used as the sole source for disease-specific mechanistic claims. Evidence was stratified by disease context: findings obtained in HCC or CRC models or patient samples were classified as disease-specific, whereas data generated in other tumor types or non-malignant systems were designated as contextual and were not extrapolated as proof of the same mechanism in HCC or CRC.

For every study contributing to a proposed H19–PD-L1 relationship, the following extraction criteria were applied:

- tumor type and experimental model;
- method of H19 perturbation (if any);
- direct measurement of CD274 messenger RNA;
- measurement of total and/or surface PD-L1 protein;
- identification of the proposed intermediate molecule (microRNA or signaling protein);
- presence of rescue or epistasis experiments;
- immune-functional readouts (T-cell activation, cytotoxicity, cytokine production);
- in vivo validation; and.
- human clinical or tissue validation.

Evidence was subsequently classified into four predefined levels:

1. Direct/experimentally demonstrated (H19 perturbation alters CD274/PD-L1 and, where claimed, immune function in the same HCC or CRC model);
2. Disease-specific component evidence (HCC- or CRC-specific findings that establish individual biological components but do not demonstrate an H19–PD-L1 connection);
3. Contextual evidence (findings from other cancers or non-malignant models);
4. Hypothetical/proposed integrated mechanism (assembled from separately reported observations without direct testing of the complete causal chain).

This hierarchical framework was applied consistently throughout the manuscript to determine wording, figure design, table construction, and conclusions.

### Evidence assessment and mechanistic classification

#### ceRNA mechanisms and PD-L1 regulation

**Fig. 1:**
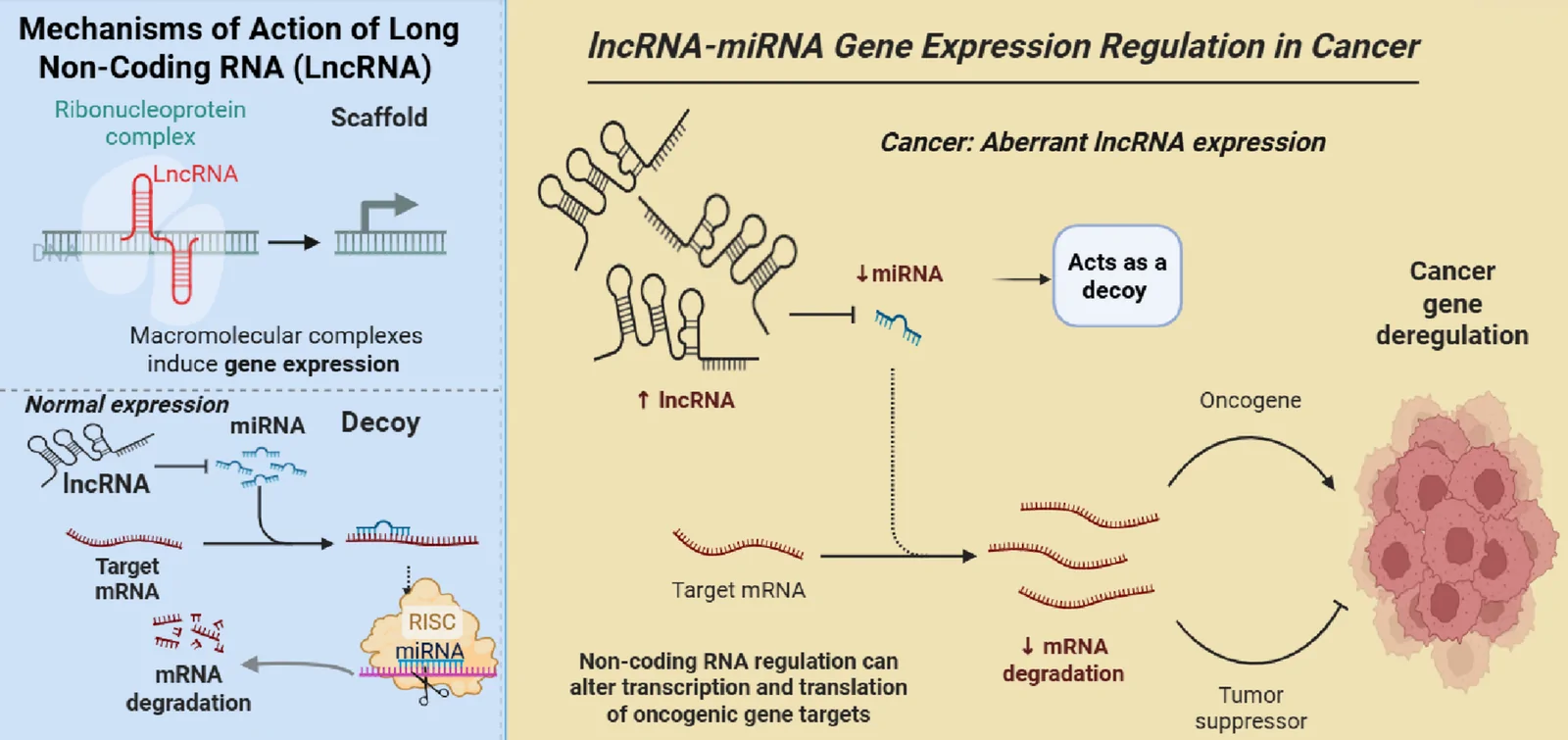
General mechanisms of lncRNA-mediated gene regulation. lncRNAs can regulate gene expression through scaffold-dependent interactions with proteins, DNA, and RNA, thereby influencing chromatin organization, transcriptional programs, and signaling complexes. lncRNAs can also function as competing endogenous RNAs (ceRNAs) by binding miRNAs and reducing miRNA availability for target messenger RNAs. Under physiological conditions, miRNAs associate with the RNA-induced silencing complex and suppress target messenger RNAs through translational repression and/or RNA degradation. This figure illustrates general lncRNA regulatory principles and does not depict an experimentally validated H19–miRNA–PD-L1 mechanism in HCC or CRC

Among the cited studies evaluated in this section, no study experimentally tested whether H19 manipulation alters CD274 messenger RNA, PD-L1 protein expression or stability, or PD-L1-associated immune function in HCC or CRC. Accordingly, an H19-dependent ceRNA mechanism regulating PD-L1 in HCC or CRC remains unestablished within the cited evidence base [15–17]. In HCC, restoration of miR-140-5p reduced doxorubicin resistance and malignant cellular phenotypes, supporting a tumor-suppressive role for miR-140-5p in this disease context [18]. However, the study did not assess H19, CD274/PD-L1, or immune-cell function and therefore does not demonstrate an H19/miR-140-5p/PD-L1 mechanism [18]. The available HCC studies establish H19-related oncogenic mechanisms but do not connect H19 to PD-L1 regulation. In HepG2 cells, NSUN2 perturbation altered m^5 C modification and stability of H19, while restoration of NSUN2 partially restored H19 methylation and abundance [15]. The study further demonstrated an interaction between m^5 C-modified H19 and G3BP1 and linked this interaction to MYC accumulation; HCC tissues also showed increased H19 expression and m^5 C methylation associated with poor differentiation [15]. These findings provide disease-specific experimental evidence for an NSUN2/m^5 C/H19/G3BP1-related HCC mechanism, but CD274/PD-L1 and immune endpoints were not assessed [15]. Separately, H19 was reported to increase miR-675 expression and to contribute to sorafenib resistance and EMT-associated phenotypes in HCC cells [16]. This study provides direct evidence for an H19-associated mechanism in sorafenib-resistant HCC cells, but it does not provide evidence for PD-L1/CD274 regulation because PD-L1-related endpoints and immune-functional outcomes were not assessed [16]. CRC provides experimentally demonstrated examples showing that ceRNA-related mechanisms can regulate PD-L1 without involving H19. Tumor-cell-derived exosomal KCNQ1OT1 sequestered miR-30a-5p, increased USP22, reduced PD-L1 ubiquitination, and promoted immune escape in CRC models [19]. This study therefore demonstrates a ceRNA-associated mechanism of post-translational PD-L1 stabilization in CRC, but it does not assess H19 [19]. Similarly, miR-124-3p directly targeted the PD-L1 3′ untranslated region, reduced PD-L1 messenger RNA, protein, and surface expression, and suppressed STAT3 signaling in CRC cells [20]. The study additionally evaluated Treg-related effects in coculture models, supporting an immune-functional consequence of miR-124-3p-mediated PD-L1 regulation in CRC [20]. However, the study did not assess H19 binding to miR-124-3p or H19-dependent regulation of the miR-124-3p/STAT3/PD-L1 pathway [20]. In microsatellite-stable CRC, IL-17 A increased PD-L1 expression through the p65/NRF1/miR-15b-5p pathway, whereas miR-15b-5p directly regulated the PD-L1 3′ untranslated region [21]. IL-17 A blockade enhanced the antitumor activity of anti-PD-1 treatment in CRC models and was associated with altered intratumoral immune-cell populations [21]. This represents direct PD-L1-specific and immune-functional evidence in MSS CRC, but H19 was not assessed [21]. Findings from other disease contexts should be interpreted only as contextual evidence. In ovarian cancer, H19 overexpression reduced miR-140-5p expression and promoted PI3K/AKT-associated invasion, migration, and EMT phenotypes [22]. Although this study supports an H19/miR-140-5p-related mechanism in ovarian cancer, it did not assess PD-L1/CD274 or immune function and cannot be extrapolated to HCC or CRC [22]. In a non-malignant osteochondral model, exosomal H19 acted as a ceRNA for miR-29b-3p, and the H19–miR-29b-3p interaction was experimentally validated using reporter, RNA pull-down, RIP, and exosome-transfer experiments [23]. Because the study involved chondrocytes and osteochondral repair rather than HCC or CRC, and did not assess PD-L1/CD274, STAT3, or tumor immunity, it represents contextual evidence only [23]. Additional contextual studies show that non-coding RNAs can regulate PD-L1 in other tumor types. In cervical cancer, LSD1 silencing was evaluated in a p53/miR-34a-associated context and in combination with anti-CD47/PD-L1 immunotherapy [24]. In triple-negative breast cancer, the miR-194/XIST/PD-L1 regulatory loop was evaluated in the context of oleuropein treatment [25]. In epithelial ovarian cancer, miR-200c-3p directly targeted the PD-L1 3′ untranslated region and reduced PD-L1 expression while modulating β-catenin and c-Myc signaling [26]. These studies support the general plausibility of non-coding-RNA-mediated PD-L1 regulation but do not establish an H19-dependent PD-L1 mechanism in HCC or CRC [24–26]. Overall, the cited studies demonstrate H19-related mechanisms in HCC and CRC and multiple PD-L1-regulatory mechanisms in HCC and CRC; however, they do not experimentally connect H19 to PD-L1/CD274 in either disease (Fig. 1) [15–17, 19–21, 27–34].

#### Tumor-suppressive lncRNAs and non-ceRNA mechanisms relevant to PD-L1 regulation

MEG3 and GAS5 should not be described as PD-L1 regulators in HCC or CRC on the basis of the cited evidence. In HCC, MEG3 suppressed proliferation, migration, and invasion through the miR-544b/BTG2 axis [35]. This study supports a tumor-suppressive MEG3-associated mechanism in HCC but did not assess CD274 messenger RNA, PD-L1 protein, PD-L1 stability, or immune-cell function [35]. In CRC, GAS5 was experimentally investigated as a tumor-suppressive lncRNA that bound miR-21, increased LIFR expression, and inhibited proliferation, migration, and metastasis-associated phenotypes [36]. The GAS5–miR-21 interaction and miR-21–LIFR relationship were evaluated using reporter-based experiments and functional rescue approaches [36]. However, Ref. 23 did not assess CD274, PD-L1 protein, PD-L1 stability, or PD-1/PD-L1-associated immune function and therefore cannot support a GAS5–PD-L1 mechanism in CRC [36]. Thus, based on the cited evidence, PD-L1 regulation by MEG3 or GAS5 in HCC or CRC remains unestablished rather than disproven [35, 36]. Several experimentally demonstrated PD-L1-regulatory mechanisms in HCC and CRC are independent of H19, MEG3, and GAS5. In HCC, WSX1 suppressed PI3Kδ/AKT signaling, promoted GSK3β-associated PD-L1 degradation, and enhanced antitumor CD8^+ T-cell activity [27]. This study provides direct evidence for a WSX1/PI3Kδ/AKT/GSK3β/PD-L1 mechanism in HCC, but it did not test H19, MEG3, or GAS5 [27]. In CRC, extracellular-vesicle-derived miR-15a reduced PD-L1 expression through the KDM4B/HOXC4 axis and increased susceptibility of CRC cells to NK-cell-mediated lysis [28]. This finding provides direct evidence for a non-ceRNA RNA-mediated mechanism of PD-L1 repression and immune-evasion attenuation in CRC, but it does not establish involvement of H19, MEG3, or GAS5 [28]. Therefore, tumor-suppressive lncRNAs and RNA-mediated pathways may represent candidates for future PD-L1-focused studies, but the cited evidence does not justify describing MEG3 or GAS5 as experimentally validated regulators of PD-L1 in HCC or CRC [28, 35, 36]. The evidence levels and appropriate interpretations of all molecular axes discussed in this section are summarized in Table 1.

**Table 1:** Evidence Classification of H19-Related Mechanisms and PD-L1-Regulatory Pathways

Molecular axis	Cancer/model context	Experimentally demonstrated finding	H19-specific evidence	PD-L1/CD274-specific evidence	H19–PD-L1 mechanistic evidence	Immune-functional evidence	Evidence classification	Appropriate interpretation	Citations
miR-140-5p	HCC	miR-140-5p restoration reduced doxorubicin resistance and malignant phenotypes.	Not assessed	Not assessed	Not assessed	Not assessed	Disease-specific component evidence	Supports a tumor-suppressive role for miR-140-5p in HCC but does not demonstrate H19 involvement or PD-L1 regulation.	[1, 18]
NSUN2/m^5 C/H19/G3BP1	HCC; HepG2 cells, xenografts, and human HCC tissues	NSUN2-dependent m^5 C modification increased H19 stability; m^5 C-modified H19 bound G3BP1 and was linked to MYC accumulation.	Demonstrated; H19 was measured, and its stability and m^5 C status were assessed after NSUN2 perturbation	Not assessed	Not assessed	Not assessed	Disease-specific component evidence	Demonstrates an NSUN2/m^5 C/H19/G3BP1-related HCC mechanism but provides no PD-L1 or immune-regulatory evidence.	[2, 15]
H19/miR-675	Sorafenib-resistant HCC cells	H19 increased miR-675 expression and contributed to sorafenib resistance and EMT-associated phenotypes.	Demonstrated	Not assessed	Not assessed	Not assessed	Disease-specific component evidence	Demonstrates H19-associated drug-resistance and EMT biology in HCC but does not demonstrate PD-L1 regulation.	[3, 16]
WSX1/PI3Kδ/AKT/GSK3β/PD-L1	HCC	WSX1 inhibited PI3Kδ/AKT signaling, promoted GSK3β-dependent PD-L1 degradation, and enhanced CD8^+ T-cell activity.	Not assessed	Demonstrated	Not assessed	Demonstrated	Direct/experimentally demonstrated evidence	Demonstrates a PD-L1-regulatory and immune-functional mechanism in HCC without evidence of H19 involvement.	[14, 27]
TNF-α/IL-6/NF-κB/STAT3/PD-L1	Hepatoma/HCC-related models	Adipocyte-derived TNF-α and IL-6 induced PD-L1 expression, and NF-κB or STAT3 inhibition reduced PD-L1 induction.	Not assessed	Demonstrated	Not assessed	Not assessed	Direct/experimentally demonstrated evidence	Demonstrates inflammatory PD-L1 induction in the reported model but does not test H19.	[16, 29]
GOLM1/EGFR/STAT3/PD-L1	HCC	GOLM1 increased EGFR-dependent STAT3 phosphorylation and PD-L1 transcription.	Not assessed	Demonstrated	Not assessed	Not assessed	Direct/experimentally demonstrated evidence	Demonstrates STAT3-associated PD-L1 transcriptional regulation in HCC without evidence of H19 involvement.	[17, 30]
IFN-γ-associated signaling/PD-L1	HCC-related model	Pentamethylquercetin inhibited adipocyte-induced PD-L1 expression in association with IFN-γ signaling.	Not assessed	Assessed	Not assessed	Not assessed	Disease-specific component evidence	Supports PD-L1 regulation in an HCC-related model but does not assess H19.	[18, 31]
DEHP/JAK2/STAT3/PD-L1	HCC models	DEHP increased PD-L1 expression through JAK2/STAT3 signaling and was associated with an immune-evasion phenotype.	Not assessed	Demonstrated	Not assessed	Reported	Direct/experimentally demonstrated evidence	Demonstrates JAK2/STAT3-associated PD-L1 induction in HCC models without testing H19.	[19, 32]
KCNQ1OT1/miR-30a-5p/USP22/PD-L1	CRC	Exosomal KCNQ1OT1 sequestered miR-30a-5p, increased USP22, reduced PD-L1 ubiquitination, and promoted immune escape.	Not assessed	Demonstrated	Not assessed	Demonstrated	Direct/experimentally demonstrated evidence	Validated ceRNA-associated post-translational PD-L1 stabilization in CRC; it is not evidence for H19.	[5, 19]
miR-124-3p/STAT3/PD-L1	CRC	miR-124-3p directly targeted PD-L1, reduced PD-L1 messenger RNA, protein, and surface expression, and suppressed STAT3 signaling.	Not assessed	Demonstrated	Not assessed	Demonstrated in Treg coculture experiments	Direct/experimentally demonstrated evidence	Demonstrates a miRNA/STAT3/PD-L1 mechanism in CRC but does not test H19.	[6, 20]
IL-17 A/p65/NRF1/miR-15b-5p/PD-L1	MSS CRC cells, murine models, and patient samples	IL-17 A increased PD-L1 through p65/NRF1-mediated repression of miR-15b-5p; IL-17 A blockade enhanced anti-PD-1 efficacy.	Not assessed	Demonstrated	Not assessed	Demonstrated	Direct/experimentally demonstrated evidence	Demonstrates an inflammatory miRNA-mediated PD-L1 and anti-PD-1-resistance mechanism in MSS CRC without H19 involvement.	[7, 21]
miR-15a/KDM4B/HOXC4/PD-L1	CRC	Extracellular-vesicle-derived miR-15a reduced PD-L1 through KDM4B/HOXC4 signaling and increased susceptibility to NK-cell-mediated lysis.	Not assessed	Demonstrated	Not assessed	Demonstrated	Direct/experimentally demonstrated evidence	Validates RNA-mediated PD-L1 repression and attenuation of immune evasion in CRC without evidence for H19, MEG3, or GAS5 involvement.	[15, 28]
LIF/PI3K/AKT/H19/SOX2	CRC cells	LIF activated PI3K/AKT, increased H19 expression, and enhanced spheroid formation and SOX2 expression; H19 knockdown attenuated these effects.	Demonstrated	Not assessed	Not assessed	Not assessed	Disease-specific component evidence	Demonstrates H19-associated stemness biology in CRC cells but does not assess PD-L1/CD274 or immune function.	[17, 20]
CCL5/p65/STAT3/CSN5/PD-L1	CRC	Macrophage-derived CCL5 induced CSN5 through p65/STAT3 signaling, stabilized PD-L1, and reduced T-cell-mediated killing.	Not assessed	Demonstrated	Not assessed	Demonstrated	Direct/experimentally demonstrated evidence	Demonstrates inflammatory post-translational PD-L1 regulation and immune escape in CRC without H19 assessment.	[21, 33]
PGE2/miR-675-5p/p53	CRC	PGE2 induced miR-675-5p and promoted metastasis through p53-associated regulation.	H19 not assessed	Not assessed	Not assessed	Not assessed	Disease-specific component evidence	Supports miR-675-5p-associated oncogenic signaling in CRC but does not establish H19 activity or PD-L1 regulation.	[22, 33]
MEG3/miR-544b/BTG2	HCC	MEG3 inhibited proliferation, migration, and invasion through the miR-544b/BTG2 axis.	Not applicable; MEG3 was experimentally investigated	Not assessed	Not assessed	Not assessed	Disease-specific component evidence	Supports tumor-suppressive MEG3 biology in HCC but does not demonstrate PD-L1 regulation.	[13, 35]
GAS5/miR-21/LIFR	CRC	GAS5 bound miR-21, increased LIFR expression, and suppressed CRC growth and metastatic phenotypes.	Not applicable; GAS5 was experimentally investigated	Not assessed	Not assessed	Not assessed	Disease-specific component evidence	Supports a GAS5/miR-21/LIFR mechanism in CRC but does not support a GAS5–PD-L1 mechanism.	[23, 36]
H19/miR-140-5p/PI3K/AKT	Ovarian cancer	H19 overexpression reduced miR-140-5p expression and promoted PI3K/AKT-associated invasion, migration, and EMT.	Demonstrated	Not assessed	Not demonstrated in HCC/CRC	Not assessed	Contextual evidence	Demonstrates an H19/miR-140-5p-related mechanism in ovarian cancer only and cannot be interpreted as HCC/CRC evidence.	[8, 22]
H19/miR-29b-3p/FoxO3	Non-malignant osteochondral model	Exosomal H19 acted as a ceRNA for miR-29b-3p, with interaction validated by reporter, RIP, RNA pull-down, and exosome-transfer experiments.	Demonstrated	Not assessed	Not demonstrated in HCC/CRC	Not assessed	Contextual evidence	Provides validated non-malignant evidence for an H19–miRNA interaction but no evidence for PD-L1 or gastrointestinal cancer biology.	[9, 23]
p53/miR-34a/PD-L1	Cervical cancer	LSD1 silencing was evaluated in a p53/miR-34a-associated context and in combination with anti-CD47/PD-L1 immunotherapy.	Not assessed	Assessed in another cancer context	Not demonstrated in HCC/CRC	Studied in an immunotherapy context	Contextual evidence	Contextual ncRNA- and PD-L1-related evidence in cervical cancer; it is not evidence for H19 in HCC or CRC.	[10, 24]
miR-194/XIST/PD-L1	Triple-negative breast cancer	The miR-194/XIST/PD-L1 regulatory loop was evaluated in the context of oleuropein treatment.	Not assessed	Assessed in another cancer context	Not demonstrated in HCC/CRC	Studied in an immune-oncology context	Contextual evidence	Contextual ncRNA/PD-L1 evidence in breast cancer; it does not support H19-dependent PD-L1 regulation in HCC or CRC.	[11, 25]
miR-200c-3p/PD-L1	Epithelial ovarian cancer	miR-200c-3p directly targeted the PD-L1 3′ untranslated region and reduced PD-L1 expression while modulating β-catenin and c-Myc signaling.	Not assessed	Demonstrated in another cancer context	Not demonstrated in HCC/CRC	Not clearly assessed	Contextual evidence	Contextual evidence for miRNA-mediated PD-L1 repression in ovarian cancer; H19 was not investigated.	[12, 26]
Integrated H19–STAT3/NF-κB/PI3K/AKT–PD-L1 model	HCC and CRC	H19-associated mechanisms and PD-L1-regulatory pathways were reported separately in different studies.	Demonstrated in separate H19-focused studies	Demonstrated in separate PD-L1-focused studies	Not demonstrated	Variable across component studies	Hypothetical/proposed integrated mechanism	The integrated model requires direct testing in the same HCC or CRC system and cannot be inferred from pathway overlap.	[15–17, 19–21, 27–34]

“Not assessed” indicates that the cited study did not report evaluation of that endpoint. “Not demonstrated” indicates that the cited evidence does not establish the stated mechanistic relationship; it does not mean that the relationship has been experimentally disproven. “Contextual evidence” denotes findings from another cancer type or a non-malignant model and should not be interpreted as HCC- or CRC-specific mechanistic evidence. “Disease-specific component evidence” supports one or more components of a proposed model in HCC or CRC but does not establish an H19–PD-L1 mechanism. “Hypothetical/proposed integrated mechanism” denotes a pathway assembled from separately demonstrated components that has not been directly tested as a continuous causal mechanism

### Signaling-pathway integration

**Fig. 2:**
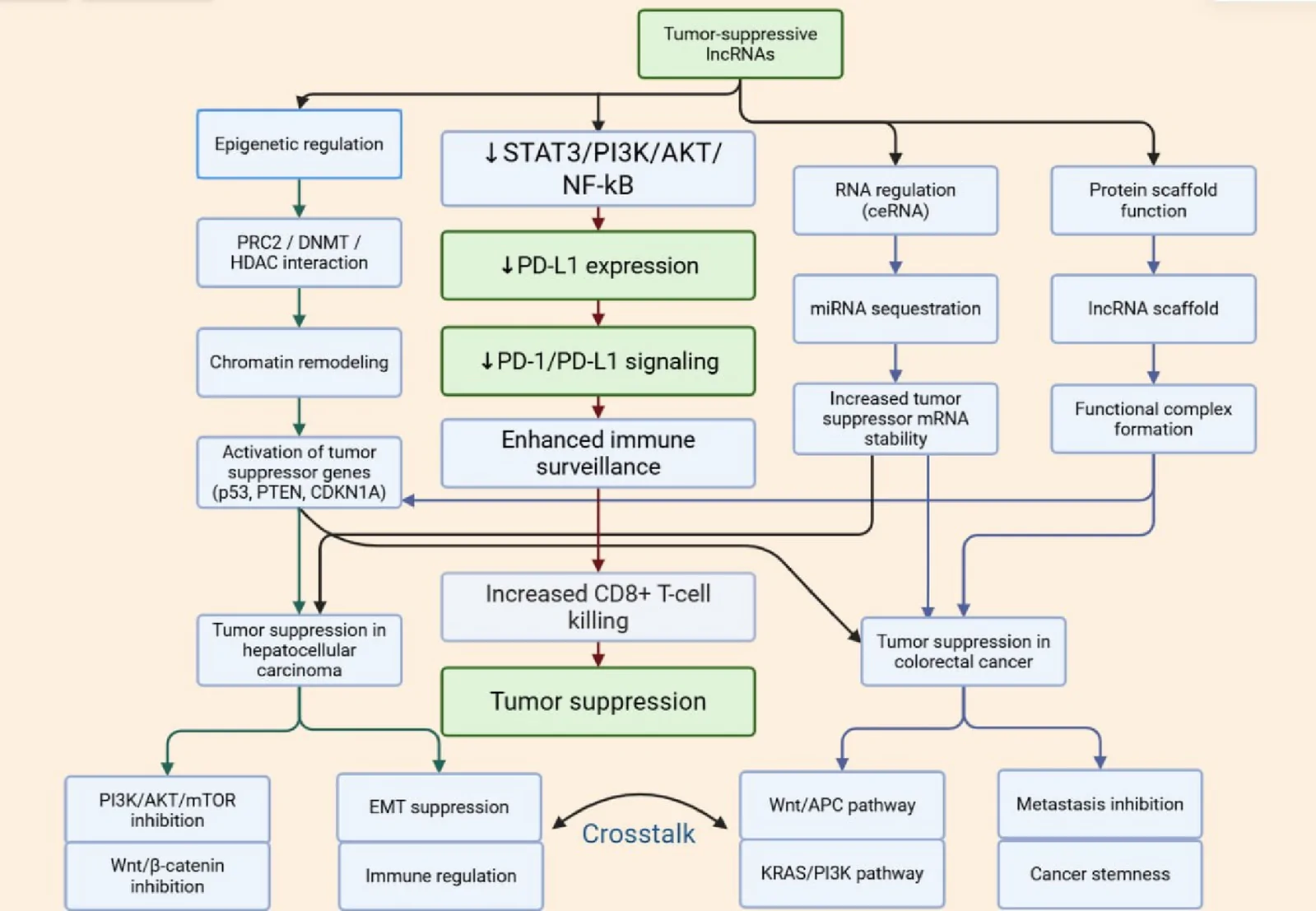
Conceptual framework for lncRNA-associated and PD-L1-regulatory signaling in HCC and CRC. Solid arrows should depict only molecular relationships experimentally demonstrated in the indicated disease/model, including WSX1/PI3Kδ/AKT/GSK3β-mediated PD-L1 regulation in HCC, TNF-α/IL-6/NF-κB/STAT3-associated PD-L1 induction in hepatoma/HCC-related models, GOLM1/EGFR/STAT3-mediated PD-L1 transcription in HCC, IL-17 A/p65/NRF1/miR-15b-5p-mediated PD-L1 regulation in MSS CRC, and CCL5/p65/STAT3/CSN5-associated PD-L1 stabilization in CRC [21, 27, 29, 30, 33]. Dashed arrows should indicate proposed or untested links between H19, MEG3, GAS5, signaling pathways, and PD-L1. The figure should not contain a solid H19→PD-L1, MEG3→PD-L1, or GAS5→PD-L1 arrow because these relationships have not been experimentally demonstrated in the cited HCC or CRC studies [15–17, 35, 36]

Several HCC studies experimentally demonstrate that inflammatory and oncogenic signaling pathways regulate PD-L1 independently of H19 (Fig. 2).

In hepatoma/HCC-related models, adipocyte-derived TNF-α and IL-6 increased PD-L1 expression, whereas pharmacological inhibition of NF-κB or STAT3 reduced this effect [29]. This study supports TNF-α/IL-6/NF-κB/STAT3-associated PD-L1 induction in the reported model, but it did not investigate H19 [16, 29]. GOLM1 enhanced EGFR-dependent STAT3 phosphorylation and increased PD-L1 transcription in HCC cells [30]. This finding provides direct evidence for a GOLM1/EGFR/STAT3/PD-L1 pathway in HCC, but it does not establish an H19–STAT3–PD-L1 mechanism [30]. Pentamethylquercetin inhibited adipocyte-induced PD-L1 expression in HCC-related experiments in association with IFN-γ signaling, whereas DEHP increased PD-L1 expression through JAK2/STAT3 signaling in HCC models [31, 32]. Neither study assessed H19; consequently, these findings constitute PD-L1-specific signaling evidence rather than evidence for H19-dependent PD-L1 regulation [31, 32]. The PI3K/AKT pathway is also implicated in PD-L1 stability in HCC. WSX1 inhibited PI3Kδ/AKT signaling and promoted GSK3β-dependent PD-L1 degradation in HCC models [27]. However, the cited HCC studies do not show that H19 regulates PD-L1 through PI3K/AKT activation or through interference with GSK3β-dependent PD-L1 degradation [15, 16, 27]. Therefore, an H19/PI3K/AKT/GSK3β/PD-L1 cascade in HCC is a hypothetical model requiring direct testing rather than an experimentally established pathway [15, 16, 27]. In CRC, LIF increased H19 expression through PI3K/AKT signaling and enhanced spheroid formation and expression of stemness-associated markers, including SOX2, in HCT116 and Caco-2 cells [17]. H19 knockdown attenuated the LIF-associated increases in spheroid formation and SOX2 expression [17]. This study provides direct evidence for LIF/PI3K/AKT/H19-associated stemness biology in CRC cells, but CD274, PD-L1 protein, T-cell activity, and immune-checkpoint response were not assessed [17]. Macrophage-derived CCL5 promoted CRC immune escape through p65/STAT3-dependent CSN5 induction, resulting in PD-L1 stabilization and reduced T-cell-mediated tumor-cell killing [33]. This study provides direct evidence for an inflammatory p65/STAT3/CSN5/PD-L1 pathway in CRC, but H19 was not investigated [33]. In another CRC study, PGE2 induced miR-675-5p and promoted metastasis through p53-associated regulation [34]. Because H19 itself, PD-L1/CD274, and immune-checkpoint outcomes were not assessed in that study, miR-675-5p should not be interpreted as evidence of an H19-dependent PD-L1 mechanism [34]. Collectively, the cited literature independently supports H19-associated oncogenic, drug-resistance, and stemness-related mechanisms in HCC and CRC, as well as STAT3-, NF-κB-, PI3K/AKT-, GSK3β-, CSN5-, and miRNA-related regulation of PD-L1 in defined HCC or CRC contexts [15–17, 19–21, 27–34]. However, among the cited studies evaluated here, the direct functional intersection between H19 and these PD-L1-regulatory pathways has not been experimentally demonstrated [15–17, 27, 29–34]. Direct validation would require H19 gain- and loss-of-function experiments in HCC or CRC models, quantification of CD274 messenger RNA and PD-L1 protein, pathway-specific rescue experiments, and immune-functional testing when immune escape is claimed.

## Effects on the Tumor Microenvironment (TME)

H19 has been implicated in remodeling the tumor immune microenvironment through several distinct mechanisms: a proposed contribution to PD-L1-mediated CD8⁺ T-cell exhaustion, exosome-mediated shaping of tumor-associated macrophage (TAM) polarization, and involvement in pre-metastatic niche conditioning [37, 38]. The strength of direct evidence varies across these axes and across tumor context (CRC versus HCC), a distinction maintained throughout this section.

### T Cell exhaustion and apoptosis

Elevated expression of H19 has been proposed to enhance the surface expression of PD-L1 in tumor cells via its ceRNA network or through the activation of the upstream oncogenic signaling. Broadly, the subsequent overexpression of PD-L1 initiates an inhibitory cascade by binding PD-1 receptors on infiltrating CD8 + T cells. This interaction alters lymphocyte biology through several downstream effects, forcing functional T cells into an exhausted, non-responsive state [39]. Similar to the general PD-1/PDL-1 interaction in tumor models, the physical binding of tumor PD-L1 to the PD-1 receptor on CD8 + cytotoxic T cells induces conformational changes in the cytoplasmic tail of PD-1. This is followed by the phosphorylation of its immunoreceptor tyrosine-based inhibitory motif (ITIM) and immunoreceptor tyrosine-based switch motif (ITSM). This results in the recruitment of the tyrosine phosphatases responsible for the dephosphorylation of proximal T-cell receptor (TCR) signaling elements, specifically targeting ZAP70, CD3ζ, and the downstream PI3K/AKT/mTOR pathways [40]. Loss of these crucial activation cascades causes T cells engaged by this axis to lose their ability to proliferate, form an immunological synapse, or execute cytotoxic granule exocytosis (e.g., perforin and granzyme B release) [41].

Persistent antigen exposure sustains calcineurin-dependent NFAT nuclear translocation, while limited costimulatory signaling constrains productive NFAT–AP-1 cooperation; this NFAT/AP-1 imbalance has been associated with induction of the IL-10 promoter and, where IL-10 is secreted into the TME, with paracrine and autocrine suppression of dendritic cell antigen presentation [42]. Parallel to the cytokine shift, the persistent PD-1/PD-L1 interaction is accompanied by elevated expression of Transforming Growth Factor-beta (TGF-β) [43].

Elevated levels of TGF-β act as a chemical driver within the TME, by activating resident fibroblasts and stimulating collagen deposition; TGF-β promotes progressive extracellular matrix remodeling and increased stromal stiffness, processes that are mechanistically indirect but well-documented downstream of sustained TGF-β signaling in HCC and CRC stroma [44, 45]. Furthermore, TGF-β signals affect the CD8 + T cells themselves, causing apoptotic induction by downregulating the anti-apoptotic protein Bcl-2 while upregulating pro-apoptotic proteins like Bim. These effects may contribute to the progressive loss or attrition of tumor-reactive T cells under TGF-β-rich conditions and may facilitate their transition toward an apoptotic state in the context of sustained signaling [46].

CD8 + T-cell exhaustion is now recognized as a heterogeneous continuum rather than a single fixed endpoint, spanning a TCF1 + progenitor-exhausted (T_PEX) population that retains self-renewal and proliferative capacity through to a terminally exhausted (T_EX) phenotype, with transition between states shaped by the intensity and duration of antigen exposure [42]. The terminally exhausted pole of this continuum is marked by upregulation of compensatory alternative immune checkpoints: driven by chronic antigen exposure, core transcription factors like TOX and Eomes remodel the T cell chromatin landscape, driving high surface expression of TIM-3, which binds Galectin-9 to act as a second, independent molecular brake alongside the primary PD-1/PD-L1 blockade [42]. Under persistent antigen exposure, CD8 + T cells at this stage additionally co-express LAG-3, which is structurally homologous to CD4 and binds MHC class II with markedly higher affinity than the native T cell coreceptor. Although LAG-3 co-expression is best established on CD4⁺ regulatory T cells, its induction on CD8⁺ cytotoxic T cells under chronic antigen exposure has been documented in HCC and CRC tumor-infiltrating lymphocyte populations, and co-expression of LAG-3 and PD-1 on the same CD8 + T cell marks a deeper, more epigenetically fixed exhaustion state than single-agent checkpoint blockade [47, 48]. The specific influences of H19 on the T_PEX-to-T_EX transition, as opposed to these general terminal-exhaustion markers, remain an open gap for future mechanistic work.

### Macrophage polarization and TAM reprogramming

Beyond its effects on cytotoxic lymphocytes, H19 extends its immunosuppressive reach into the myeloid compartment through exosome-mediated reprogramming of tumor-associated macrophages. The M1/M2 nomenclature used throughout this section follows the field’s conventional, pedagogically convenient classification; human TAMs in reality occupy a continuum of activation states rather than two discrete phenotypes [49]. Within this framework, TAM stimulation is considered an important driver of lncRNA H19 upregulation in CRC tumor cells, creating a localized pool of H19 that stabilizes the immunosuppressive niche and constrains macrophage polarization toward an anti-tumor, M1-like phenotype. This TAM-induced H19 in turn targets the β-catenin signaling pathway, enhancing EMT and migratory/invasive capacity [49]. High H19 expression and M2-like macrophage infiltration are associated with the Consensus Molecular Subtype 4 (CMS4) of CRC, characterized by extensive stromal remodeling, angiogenesis, and immune suppression. This M2-skewed phenotype has been linked to chemoresistance in CRC patients and to expansion of the underlying colorectal cancer stem cell population [50]. More broadly, exosomal H19 derived from the CRC stroma has been directly shown to promote colorectal cancer stem cell stemness and confer chemoresistance in vitro and in vivo through activation of the same β-catenin pathway [51].

In the HCC microenvironment, CD68⁺ TAM-induced H19 upregulation drives sequestration of miR-193b, relieving post-transcriptional suppression of MAPK1 and promoting HCC cell migration, invasion, and EMT-associated aggressiveness [52]. In contrast to its association with M2-like maintenance in CRC, H19 in the hepatic context has instead been linked to a pro-inflammatory, M1-like macrophage phenotype during disease progression: upregulated H19 promotes an activated secretory state in hepatic-resident macrophages (Kupffer cells), inducing release of the pro-inflammatory mediators CCL2 (Chemokine Ligand 2) and IL-6 via CCL2/CCR2 signaling [53]. While M1-like macrophages are traditionally considered anti-tumor, in the liver this sustained H19-mediated pro-inflammatory state contributes to liver injury, tissue hyperplasia, and fibrosis — a microenvironment permissive for hepatocarcinogenesis [54].

### Exosomal H19 as a delivery vehicle

H19 is packaged into extracellular vesicles in both CRC and HCC, through distinct cellular sources, distinct vesicle populations, and distinct downstream mechanisms that should not be conflated. In CRC, H19 is loaded into small extracellular vesicles (CRC-sEVs) and acts on the hepatic pre-metastatic niche — a process reflecting the biology of CRC liver metastasis rather than primary HCC. CRC cells package H19 into these sEVs alongside the RNA-binding protein Fox-1 Homolog 2 (RBFOX2) [55]. Once taken up by distant, healthy parenchymal cells such as hepatocytes, the CRC-sEVs reprogram post-transcriptional gene expression to drive Epithelial-to-Mesenchymal Transition (EMT) and niche preparation [55, 56].

Within this CRC-sEV axis, H19 acts not through its canonical ceRNA activity but as an RNA-protein scaffold: one critical target is the pre-mRNA of the small GTPase RAC1, where H19 recruits RBFOX2 (often with hnRNPM) to force retention of exon 3b, converting the transcript into its oncogenic isoform RAC1B. In the newly primed pre-metastatic niche, RAC1B upregulation is associated with hyperactivation of c-Myc and Cyclin D, producing morpho-functional alterations that fuel EMT in the recipient hepatocytes and create a liver environment favorable for metastasis [57, 58].

A mechanistically separate axis operates in primary HCC. H19 exosomes are associated with CD90 + Liver Cancer Stem Cells (CSCs), a highly metastatic, stem-like subset of HCC cells characterized by the CD90 + surface marker. Unlike parental hepatoma cells, CD90 + liver CSCs selectively enrich lncH19 into their secreted exosomes. These exosomes travel through the local hepatic circulatory system or systemic bloodstream and are rapidly internalized by Human Umbilical Vein Endothelial Cells (HUVECs) and local stromal cells [59]. Exosomal H19 stimulates endothelial cells to increase VEGF and VEGFR1 expression, promoting blood vessel formation, and upregulates ICAM1, making blood vessels more adhesive so circulating HCC cells can attach and leave the bloodstream more easily [60]. In this HCC-CSC axis, H19 also acts through its canonical ceRNA activity: internalized exosomal H19 sponges miR-520a-3p, relieving post-transcriptional suppression of LIM Domain Kinase 1 (LIMK1). Elevated LIMK1 reorganizes the actin cytoskeleton, providing the morphological flexibility that enhances cell migration, invasion, and metastatic potential [61].

## Clinical implications and translational potential

### H19 as a diagnostic and prognostic biomarker in HCC and CRC

H19, together with Colon Cancer-Associated Transcript 1(CCAT1) and MALAT1, is significantly upregulated in the sera, Peripheral Blood Mononuclear Cells(PBMCs), and tissues of HCC patients compared with Hepatitis C Virus(HCV) patients and healthy controls, supporting further investigation of H19 as a potential diagnostic biomarker [62]. In addition to its function as a ceRNA, H19 serves as the precursor of miR-675, a distinct regulatory axis through which H19-associated effects may be mediated by the mature miRNA rather than by direct sequestration of other miRNA [63]. H19-derived miR-675 has also been investigated for its potential diagnostic relevance in HCC and as a potential therapeutic target [64]. Elevated H19 levels in HCC tissues and plasma, together with their reduction following successful treatment, suggest that H19 warrants further investigation as a circulating marker of disease presence and treatment-associated changes [65]. In CRC, H19 overexpression is associated with poorer disease-free, overall, and recurrence-free survival; however, these associations alone do not establish H19 as a clinically validated prognostic biomarker. Establishing independent prognostic value will require adequately powered cohorts, multivariable analyses accounting for established clinicopathological factors, and independent validation [66]. Circulating EV-associated H19/miR-675 has been investigated as a minimally invasive diagnostic marker and has also shown associations with clinical outcomes. Separately, H19 SNPs have been associated with treatment response; however, such associations should not be interpreted as evidence of a clinically validated predictive biomarker without demonstration of differential treatment benefit [67].

Although the primary focus of this review is HCC and CRC, findings from other malignancies provide contextual evidence supporting the broader feasibility of circulating H19 as a cancer biomarker. Sequencing studies have identified H19 and other lncRNAs as differentially expressed in lung cancer, suggesting their potential diagnostic relevance [68]. Technical and preanalytical factors should also be considered when evaluating circulating H19 as a clinical biomarker. Measurements obtained from plasma, serum, peripheral blood mononuclear cells (PBMCs), or extracellular vesicles represent distinct analytes and may not be directly comparable. Differences in sample collection and processing, hemolysis, RNA extraction and normalization procedures, extracellular vesicle isolation methods, and assay platforms may contribute to variability in measured H19 levels. Standardization of preanalytical and analytical procedures, together with independent validation across patient cohorts and assay platforms, will therefore be important for establishing the reproducibility and clinical utility of circulating H19.

However, current evidence remains insufficient to establish H19 as a clinically validated diagnostic or prognostic biomarker. In HCC, clinically relevant diagnostic evaluation frequently requires discrimination from cirrhosis or other chronic liver diseases rather than comparison with healthy individuals alone. Future studies should therefore evaluate the ability of H19 to distinguish HCC from these high-risk populations while accounting for potential confounding effects of disease etiology, liver function, inflammatory activity, tumor burden, and treatment [69].

### H19, immune regulation, and potential relevance to immunotherapy

In HCC, the phase III IMbrave150 trial demonstrated improved overall and progression-free survival with atezolizumab (anti-PD-L1) plus bevacizumab compared with sorafenib, while the phase III HIMALAYA trial showed improved overall survival with the STRIDE regimen combining durvalumab (anti-PD-L1) and tremelimumab which is an anti- Cytotoxic T-Lymphocyte-Associated Protein 4(CTLA-4) [70, 71]. In Microsatellite Instability-High/Deficient DNA Mismatch Repair (MSI-H/dMMR) metastatic CRC, the phase III KEYNOTE-177 trial demonstrated significantly longer progression-free survival with pembrolizumab (anti-PD-1) than with chemotherapy [72]. These clinical trials establish the therapeutic relevance of immune checkpoint blockade in HCC and selected CRC populations. However, they provide no direct evidence regarding H19 and should therefore be considered clinical context for investigating molecular determinants of immune responsiveness rather than evidence supporting H19 itself.

In the liver, antigen processing and presentation contribute to the balance between immune tolerance and immune activation [73]. MHC class I presentation mediates recognition by CD8⁺ T cells, whereas MHC class II presentation supports CD4⁺ T-cell activation [74]. During hepatic inflammation such as autoimmune hepatitis, hepatocytes can aberrantly express MHC class II, while IFN-γ can enhance MHC class I expression and promote T-cell activation [75]. Thus, MHC-I/CD8⁺ and MHC-II/CD4⁺ antigen-presentation pathways should be considered alongside PD-1/PD-L1 signaling when discussing hepatic immune regulation [75, 76]. This distinction illustrates how alterations in antigen presentation can shift the hepatic immune environment from tolerance toward pathogenic immune activation, while remaining mechanistically distinct from immune escape in HCC [77].

Importantly, checkpoint blockade response depends on multiple factors beyond PD-L1 expression, including tumor antigenicity, interferon signaling, antigen processing and presentation, immune exclusion, stromal architecture, and myeloid suppression. Therefore, H19 should be evaluated within this broader immune context rather than solely through its effects on PD-L1. Across the available literature, H19 has additionally been associated with prognosis and immune-cell infiltration in colon cancer [11, 78, 79]. H19 may promote immune escape in HCC by regulating the PD-1/PD-L1 and CD155/T-cell Immunoreceptor with Ig and ITIM Domains(TIGIT) axes [80]. H19 knockdown reduces PD-L1 and CD155 co-expression and enhances tumor cell susceptibility to immune cell-mediated cytotoxicity, highlighting its potential immunoregulatory role [62, 81]. H19 may also modulate the tumor immune microenvironment, as its expression is associated with CD4 + T-cell and macrophage infiltration and poorer overall survival [78]. H19 knockdown suppresses Cyclin D1(CCND1) and VEGFA, which are positively associated with macrophage infiltration [82]. Extracellular vesicle-associated H19 has additionally been implicated in macrophage polarization by increasing the expression of inflammatory and stimulatory factors, including CXCL10 and TNF-α [83].

In CRC, the relationship between H19 and immunotherapy response is less directly established. H19 has been associated with prognosis and immune-cell infiltration in colon cancer [11, 84, 85]. In CRC, miR-138-5p has been reported to suppress tumor immune escape by directly targeting PD-L1, suggesting its potential relevance to immune checkpoint regulation and antitumor immunity [86](Fig. 2). Direct evidence linking H19 to clinical responses to immune checkpoint inhibitors remains limited. However, H19-mediated regulation of tumor progression, stemness, autophagy, and drug resistance may contribute to an immunosuppressive tumor microenvironment in CRC [87], highlighting the potential intersection between H19-mediated cellular resistance mechanisms and antitumor immunity. In CRC, the potential relevance of H19 to immunotherapy should also be considered in the context of microsatellite instability (MSI) and mismatch repair (MMR) status, which are established determinants of response to immune checkpoint blockade [72]. Therefore, associations between H19 expression and immune-cell infiltration in unselected CRC populations cannot be directly interpreted as evidence that H19 predicts benefit from PD-1/PD-L1 blockade [88]. Future studies evaluating H19 as a potential biomarker of immunotherapy response should therefore stratify patients according to MSI/MMR status and consider tumor location, metastatic site, treatment line, and other established clinical determinants of treatment response.

Current evidence linking H19 to immunotherapy response remains largely mechanistic and preclinical, with studies demonstrating its involvement in the regulation of immune checkpoints, including PD-L1 and CD155 [62]. However, H19 has not yet been clinically validated as a predictive biomarker for immune checkpoint inhibitor response [89]. Future clinical studies should evaluate whether tissue or circulating H19 levels correlate with treatment response, survival outcomes, and resistance to immune checkpoint blockade [90].

### H19-directed therapeutic strategies

Preclinical evidence has identified H19 as a candidate therapeutic target in HCC and CRC because of its involvement in tumor progression, treatment resistance, and immune regulation [91]. Importantly, the therapeutic relevance of H19 appears to arise from several distinct molecular mechanisms rather than from a single mode of action. These mechanisms include ceRNA-mediated post-transcriptional regulation, RNA–protein interactions and alternative-splicing regulation, extracellular-vesicle-mediated intercellular transfer, and modulation of downstream signaling pathways.

#### CeRNA-mediated post-transcriptional regulation

One of the major mechanisms through which H19 regulates tumor progression is its activity as a competing endogenous RNA, whereby H19 interacts with specific miRNAs and modulates the expression of their downstream target genes [92, 93]. In HCC, H19 promotes cell proliferation and suppresses apoptosis through the miR-520a-3p/LIMK1 and miR-675/p53 axes, whereas H19 knockdown inhibits tumor growth and enhances apoptosis [11]. Increased H19 can also function as a ceRNA by binding miR-520a-3p, thereby relieving its inhibitory effect on LIMK1, a kinase involved in HCC cell proliferation and metastasis [94]. The immunoregulatory functions of H19 may provide an additional rationale for H19-directed therapeutic strategies. In HCC, knockdown of H19 reduces PD-L1 and CD155 co-expression and enhances immune cell-mediated cytotoxicity [62, 81]. H19 inhibition may exert both antitumor and immunomodulatory effects, suggesting its potential combination with PD-1/PD-L1 or CD155/TIGIT checkpoint blockade. However, the therapeutic benefit of such combinations has not yet been clinically established [62]. In CRC, H19 acts as a ceRNA for miR-194-5p, promoting Forkhead Box Protein M1 (FoxM1)-mediated epithelial-to-mesenchymal transition (EMT) and thereby enhancing tumor invasion and metastasis [67]. H19 also promotes oxaliplatin resistance and cancer stemness in CRC by interacting with miR-141 and activating WNT/β-catenin signaling, with H19 expression further enhanced by hypoxia and oxaliplatin exposure [95]. Similarly, H19 promotes EMT in CRC through regulation of the miR-29b-3p/Progranulin (PGRN) axis and activation of Wnt signaling [96]. These findings illustrate the contribution of H19-mediated miRNA regulation to tumor progression and therapeutic resistance.

#### RNA–protein interactions and alternative-splicing regulation

Beyond ceRNA activity, H19 can regulate tumor-associated processes through direct or functional interactions with RNA-binding proteins and other protein partners [97]. In CRC, H19 promotes tumor growth through its interaction with eukaryotic translation initiation factor 4A3 (EIF4A3) [98]. More recently, H19 has been implicated in the regulation of alternative splicing through interactions with RNA-binding proteins. CRC cells package H19 together with the RNA-binding protein RBFOX2 into extracellular vesicles [55]. In addition, H19 interacts with RBFOX2, together with heterogeneous nuclear ribonucleoprotein M (hnRNPM), and facilitates their recruitment to RAC1 pre-mRNA. This promotes retention of exon 3b in the RAC1 transcript and increases production of the oncogenic RAC1B isoform. In the proposed pre-metastatic niche, increased RAC1B may contribute to downstream activation of c-Myc and Cyclin D and to phenotypic changes associated with EMT in hepatocytes [57, 58]. Thus, the H19/RBFOX2/RAC1B axis represents a mechanism distinct from ceRNA activity and illustrates how H19 can regulate cancer-associated phenotypes through RNA–protein interactions and alternative-splicing control.

#### Extracellular-vesicle-mediated intercellular transfer

Extracellular-vesicle-mediated transfer represents another mechanism through which H19 may contribute to intercellular communication, tumor progression, and therapeutic resistance. CRC cells can package H19 into small extracellular vesicles (sEVs), which may reach distant parenchymal cells, including hepatocytes, where their uptake and H19-associated molecular effects may contribute to pre-metastatic niche formation. In this context, H19-containing sEVs have been associated with changes in post-transcriptional gene regulation and EMT-related phenotypes in recipient hepatocytes [55, 57]. Similarly, extracellular-vesicle-associated H19 has been associated with increased stemness and chemoresistance in CRC [99]. CAF-derived extracellular-vesicle-associated H19 has also been associated with enhanced chemoresistance and stem-like properties, further supporting a role for vesicular H19 in tumor–stromal communication [90]. These findings suggest that H19 may influence cancer progression not only within tumor cells but also through intercellular transfer between tumor, stromal, and distant recipient cells.

#### Regulation of downstream signaling and therapeutic resistance

H19-mediated effects can also converge on signaling pathways and cellular processes that contribute to therapeutic resistance. H19-mediated regulation of autophagy may contribute to drug resistance in CRC [100]. In HCC, H19 has been associated with metastasis and tumor progression through regulation of the miR-193b/MAPK1 pathway and may contribute to chemoresistance by influencing multidrug resistance protein 1 (MDR1) expression and reducing sorafenib sensitivity through EMT-related mechanisms [52, 90, 101]. H19 may also promote tumor progression by activating RAS/MAPK signaling through the miR-140-5p/Son of Sevenless Homolog 1 (SOS1) axis in HCC [100]. In addition, the immunoregulatory functions of H19 provide a potential rationale for therapeutic targeting. In HCC, H19 knockdown reduces PD-L1 and CD155 co-expression and enhances immune cell-mediated cytotoxicity. Accordingly, H19 inhibition may exert both antitumor and immunomodulatory effects and could potentially be investigated in combination with PD-1/PD-L1 or CD155/TIGIT checkpoint blockade. However, the therapeutic benefit of such combinations has not yet been clinically established [80, 102](Fig. 2).

**Fig. 3:**
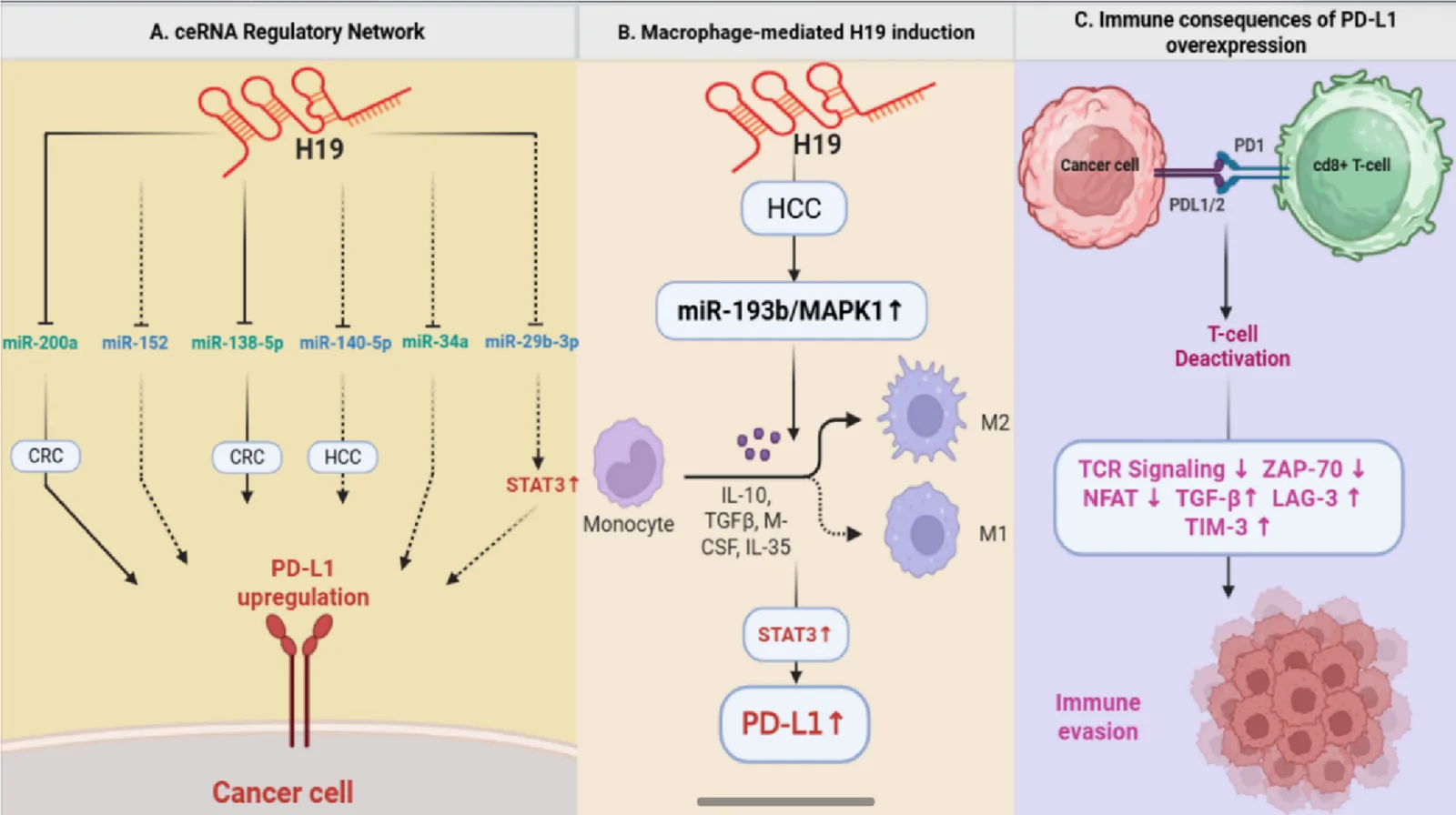
Proposed H19-associated regulatory networks potentially intersecting with PD-L1 signaling and immune regulation in hepatocellular carcinoma (HCC) and colorectal cancer (CRC). This figure summarizes experimentally supported and proposed mechanisms through which H19 may intersect with PD-L1 regulation and tumor immune responses in HCC and CRC, while distinguishing disease-specific evidence from mechanistic connections that remain indirect or hypothetical. Solid arrows indicate experimentally supported relationships within the relevant disease or experimental context, whereas dashed arrows indicate indirect, inferred, predicted or hypothesis-generating relationships; the presence of individual components within the same panel does not imply that the complete pathway has been experimentally validated. (**A**) Proposed ceRNA-related mechanisms. H19 has been reported to interact with multiple microRNAs in different experimental contexts, and several of these microRNA pathways have independently been associated with PD-L1 regulation or immune-related signaling. However, these observations do not necessarily establish a single H19–microRNA–PD-L1 pathway in HCC or CRC. Experimentally supported interactions are therefore distinguished from proposed or context-derived connections. In particular, the relationships between H19, miR-200a, miR-138-5p, miR-140-5p and other candidate microRNAs should be interpreted according to the disease model and experimental evidence supporting each interaction. Dashed arrows indicate inferred, predicted or hypothesis-generating relationships rather than experimentally established causal links. Collectively, these pathways suggest that H19 may intersect with regulatory networks controlling PD-L1 expression, but direct H19-dependent regulation of PD-L1 should not be inferred where the complete molecular pathway has not been experimentally demonstrated. (**B**) H19-associated macrophage and inflammatory signaling. H19 has been implicated in macrophage-associated and inflammatory signaling pathways in hepatic and tumor-related experimental contexts. In HCC-related studies, the H19/miR-193b/MAPK1 axis has been associated with changes in signaling and inflammatory communication, whereas cytokine-mediated macrophage polarization, STAT3 activation and PD-L1 expression represent additional components of the tumor immune microenvironment. These observations may be biologically interconnected, but the complete sequence linking H19 to cytokine production, macrophage polarization, STAT3 activation and PD-L1 induction has not necessarily been demonstrated as a single causal pathway. Accordingly, the figure presents these relationships as associated or potentially convergent mechanisms rather than as a fully validated H19-driven feedback loop. (**C**) Established consequences of PD-1/PD-L1 checkpoint engagement and their potential relevance to H19-associated immune regulation. Engagement of PD-1 by PD-L1 can attenuate T-cell receptor signaling and effector functions and, under conditions of persistent antigenic stimulation, is associated with broader states of T-cell dysfunction and exhaustion. Additional inhibitory receptors, including TIM-3 and LAG-3, and transcriptional regulators such as NFAT and TOX can participate in these dysfunctional states. These established consequences of sustained checkpoint signaling provide the biological context for considering a potential relationship between H19-associated regulation of PD-L1 and immune dysfunction. Activation of the PD-1/PD-L1 immune checkpoint suppresses T-cell receptor (TCR) signaling and downstream effector pathways, including reduced activity of ZAP-70 and relocation of nuclear factor of activated T cells (NFAT) in T cell. Concurrently, the immunosuppressive cytokine TGF-β is increased, while inhibitory immune checkpoint receptors TIM-3 and LAG-3 are upregulated, promoting a state of T-cell exhaustion. However, the figure does not imply that H19 directly controls each component of the T-cell exhaustion program. Where a direct H19-dependent connection has not been experimentally demonstrated, the relationship should be interpreted as indirect or hypothesis-generating. Overall, Fig. 3 integrates disease-specific observations with biologically plausible connections among H19-associated RNA regulation, inflammatory and macrophage-related signaling, PD-L1 biology and T-cell dysfunction. The figure is intended as an evidence-informed conceptual framework rather than a representation of a single experimentally validated H19-centered pathway

### Context-Dependent Role of H19 in HCC

H19 should not be considered intrinsically oncogenic or tumor-suppressive in HCC. Although many studies describe H19 as an oncogenic lncRNA, reduced H19 expression and tumor-suppressive or metastasis-inhibitory effects have also been reported in specific experimental and clinical contexts [99, 103]. The majority of in vivo studies provide evidence supporting a tumor-suppressive role for H19 [87, 100]. Furthermore, H19 is downregulated in HCC tumor tissues compared to peritumoral tissues and suppresses metastasis by regulating the miR-200 pathway and promoting mesenchymal to epithelial transition (MET) [103] (Fig. 2). H19 suppresses propofol-induced apoptosis in HCC cells by acting as a sponge for miR-520a-3p, thereby increasing LIMK1 expression [61]. Although H19 is frequently upregulated across various cancers, reduced H19 expression has also been observed in certain tumor types [104], highlighting its dual role in cancer. Fnd sample source, as well as the complex and heterogeneous nature of HCC, may contribute to the divergent findings reported across studies [84, 99]. In particular, H19 expression assessed in tumor tissue may not necessarily reflect its abundance or biological significance in other sample types, such as plasma or extracellular vesicles, which should be considered when evaluating its potential clinical utility [82, 99]. Several factors may account for these divergent observations. First, cellular and tissue source may influence the biological interpretation of H19 expression. H19 measured in tumor tissue or peritumoral tissue may not reflect its abundance or function in circulating compartments such as plasma or extracellular vesicles. Second, disease stage and experimental model may influence H19 expression and function, such that effects observed in early tumor development may differ from those observed during invasion or metastasis. Third, etiological and epigenetic differences may alter H19 expression and activity. H19 is an imprinted transcript whose expression is closely linked to the H19/IGF2 locus, and aberrant methylation or loss of imprinting may modify its expression during hepatocarcinogenesis [85, 105, 106]. Finally, the functional consequences of H19 may depend on the availability of interacting microRNAs and RNA-binding proteins within a particular cellular context (Table 2).

### Context-Dependent Role of H19 in CRC

H19 is predominantly characterized as an oncogenic lncRNA in CRC, although its biological effects may also vary according to cellular, molecular, and therapeutic context [67, 107]. Notably, ITF2357 also induced apoptosis in 5-fluorouracil-resistant HCT-116 cells expressing high levels of H19, further suggesting that the biological effects of H19 may depend on the therapeutic context [108]. H19 has also been implicated in 5-FU resistance through regulation of the miR-194-5p/SIRT1/autophagy pathway, further supporting its predominantly oncogenic and treatment-resistant phenotype [91]. The H19 rs2839698 polymorphism has been associated with reduced CRC risk in a Chinese Han population; however, this genetic association does not directly indicate a tumor-suppressive role for H19 [109]. Collectively, current evidence indicates that although H19 predominantly exhibits oncogenic activity in CRC, its biological and clinical significance may vary according to therapeutic, molecular, and genetic contexts [67, 108, 110] (Tables 2 and 3).

**Table 2:** Context-dependent expression and functional effects of H19 in HCC and CRC

Cancer type	H19 expression	Cellular/tissue source	Experimental model	Functional phenotype	Molecular mechanism	In vivo evidence	Reference
HCC	↑	Hep3B HCC cells; HCC tumor tissue	Hep3B cells; H19 knockdown/overexpression; xenograft model	H19 promotes tumor growth and tumorigenicity; knockdown suppresses tumor growth	H19-dependent regulation of genes involved in survival, angiogenesis, and tumorigenesis; hypoxia-responsive regulation	Yes Hep3B xenografts	[111]
HCC	↓	HCC tumor vs. peritumoral liver tissue	HCC patient tissues; SMMC7721 and HCCLM3 cells; metastasis models	H19 suppresses HCC progression, EMT, and metastasis	Activation of the miR-200 family through association with hnRNP U/PCAF/RNA Pol II and promotes MET	**Yes** metastasis models	[103]
HCC	↓	H19/Igf2 locus; liver tumor models	Genetically modified mouse models, including SV40-induced hepatocarcinoma	H19 loss accelerates tumor development, supporting a tumor-suppressive function in vivo	H19/Igf2 imprinting and locus-dependent regulation	**Yes** — murine hepatocarcinoma model	[87]
HCC	↑	HCC cells/tissues	HCC cell lines; gain- and loss-of-function experiments	H19 promotes proliferation, migration, and invasion	H19/miR-15b/CDC42/PAK1 signaling	Not established	[111]
CRC	↑	Primary CRC tissue; liver-metastatic CRC tissue	CRC cell lines; primary CRC and matched liver metastases; metastatic models	H19 promotes metastasis and is associated with poor survival	H19 interaction with hnRNPA2B1 and regulation of metastasis-associated pathways	Yes metastasis model	[112]
CRC	↑	CRC tissues and recurrent CRC samples	CRC cell lines; 5-FU-sensitive and resistant models	H19 promotes 5-FU resistance and recurrent disease phenotype	H19/miR-194-5p/SIRT1/autophagy axis	Not established	[91]
CRC	↑	CRC tumor tissue	CRC cell lines; patient cohorts	H19 promotes proliferation and migration and is associated with poorer survival	RB1–E2F and CDK8–β-catenin signaling	Not established	[113]
CRC	Context-dependent: ↑ during metastatic colonization but evidence of repression during EMT	Primary CRC, liver metastases, CRC cell models	Cellular model of CRC progression; primary tumors and liver metastases; mouse metastatic model	H19 may suppress early EMT-associated progression but promote later metastatic colonization	CpG methylation/histone modifications at H19; stage-dependent regulation	**Yes** — metastatic model	[114]

**Table 3:** Clinical Significance of H19 in HCC and CRC

Clinical application	Sample type	Main finding	Association with PD-L1/immunity	Potential utility	References
Diagnosis (HCC)	Plasma exosomes	Exosomal H19 is elevated in HCC and shows diagnostic potential for distinguishing HCC from controls	Indirect immune relevance through exosomal signaling) No direct PD-L1 analysis(	Non-invasive liquid biopsy	[115, 116]
Immune escape/checkpoint regulation (HCC)	Serum, PBMCs, liver tissue	H19 is overexpressed in hepatocellular carcinoma and influences immune checkpoint pathways, including PD-1/PD-L1 and CD155/TIGIT signaling, indicating a role in immune regulation and tumor immune escape	positive relationship between H19 activity and PD-L1-associated pathways	**Potential predictive biomarker** for response stratification in immunotherapy and candidate therapeutic target.	[62, 117]
Prognosis (HCC)	Tumor tissue/circulating RNA	Elevated H19 expression has been associated with tumor progression and unfavorable clinicopathological features in HCC, suggesting potential prognostic value	Elevated H19 expression is linked to enhanced tumor progression and may contribute to an immunosuppressive tumor microenvironment in HCC	Prognostic stratification and longitudinal disease monitoring	[62, 115]
Immune microenvironment (CRC)	Tumor tissue/circulating RNA	In colorectal cancer, H19 is overexpressed and contributes to tumor progression, epithelial mesenchymal transition, tumor microenvironment remodeling, and therapy resistance	H19 may contribute to immune-related signaling and tumor microenvironment regulation in colorectal cancer, although direct links to PD-L1 response remain limited	Candidate biomarker for future immunotherapy studies	[67]

### H19-mediated crosstalk across cancer types

Across cancer types, H19-mediated crosstalk can involve distinct mechanisms, including extracellular-vesicle-mediated transfer, RNA–protein interactions, and ceRNA activity [57]. Systems-level analyses in other malignancies, including ovarian cancer, have identified interconnected gene modules and candidate therapeutic targets, highlighting the broader complexity of cancer-associated regulatory networks. In HCC, H19 has been implicated in immune escape through regulation of PD-L1 and CD155 and modulation of macrophage-associated signaling [80](Fig. 3). In CRC, cancer-associated fibroblast (CAF)-derived extracellular vesicle-associated H19 mediates stromal–tumor communication, promoting cancer stemness and chemoresistance [51, 78]. Similarly, extracellular vesicle-mediated transfer of H19 contributes to tumor–stromal crosstalk and therapeutic resistance in breast and lung cancers [118, 119]. H19 dysregulation has also been reported in bladder cancer, where exosomal H19 has demonstrated clinical and tumor-associated relevance [120]. In glioblastoma, recent evidence has identified an H19-derived immune-related protein associated with the establishment of an immunosuppressive tumor microenvironment [121]. Increased circulating H19 has been reported in gastric and breast cancers, with levels associated with tumor-related clinical features and decreasing after tumor removal, further supporting its potential as a circulating cancer biomarker [122]. In gastric cancer, H19 has shown moderate discriminatory ability between tumor tissues and controls (AUC = 0.697) [123], while changes in circulating H19 following treatment have also been reported in breast and gastric cancers [124]. In breast cancer, H19 promotes tumor cell proliferation and invasion by sponging miR-152, thereby upregulating DNA Methyltransferase 1(DNMT1) expression and highlighting the H19/miR-152/DNMT1 regulatory axis in breast cancer progression [125] (Fig. 2). miR-34a directly targets PD-L1 and suppresses its expression, highlighting a broader role for non-coding RNA networks in regulating immune escape across different cancer types [126] (Fig. 2). Collectively, these findings suggest that H19 may participate in context-dependent communication among tumor, stromal, and immune components across multiple malignancies.

## Limitations

Several limitations should be considered when interpreting the current evidence linking H19 to immune checkpoint biology in HCC and CRC. First, the evidence base is heterogeneous across tumor types, biological compartments, experimental models, and assay platforms. Several proposed H19–PD-L1 regulatory axes, including H19/miR-34a and H19/miR-29b-3p/STAT3, are supported primarily by indirect evidence or findings from other tumor types and have yet to be directly validated in HCC and CRC [126, 127]. Tumor-specific differences in miRNA expression, signaling activity, and the immune microenvironment may influence whether these interactions are functionally relevant [128]. In addition, a substantial proportion of mechanistic evidence derives from cell-line-based and other preclinical models, which may not fully capture the cellular heterogeneity, stromal interactions, and immune complexity of human tumors. Some mechanistic interpretations also rely on secondary literature or extrapolation from related pathways rather than direct primary experimental validation, further emphasizing the need to distinguish established findings from inferred mechanisms.

Second, H19 exhibits context-dependent oncogenic and tumor-suppressive functions that may vary according to tumor stage, etiology, cellular compartment, and biological context [129]. Measurements of H19 in bulk tumor tissue may also reflect contributions from malignant, stromal, and immune cells, whereas circulating and extracellular vesicle-associated H19 represent biologically distinct compartments. Interpretation is further complicated by the organization of the imprinted H19/IGF2 locus and by the potential consequences of experimentally manipulating H19 on H19-derived miR-675 and neighboring imprinting biology. These features make it difficult to attribute observed phenotypes exclusively to H19 RNA itself and highlight the need for compartment-specific and mechanistically controlled studies.

Third, clinical biomarker studies are challenging to compare due to differences in biological material (tissue, plasma, serum, and EVs), pre-analytic processing, and assay platforms [130]. Such methodological variation may contribute to differences in reported H19 levels and associations across studies. In HCC, disease etiology and the underlying chronic liver disease may further influence H19 measurements, whereas in CRC, factors such as MSI/dMMR status, tumor location, metastatic site, and treatment context may affect their biological and clinical interpretation. Consequently, apparent differences between studies should not necessarily be interpreted as biological contradictions without accounting for differences in sample type and experimental design.

Fourth, evidence connecting H19 directly to immune-cell states and immune checkpoint inhibitor response remains incomplete. Existing studies have focused on selected immune populations and checkpoint-related pathways rather than comprehensively characterizing the cellular and functional complexity of the tumor immune microenvironment [80]. Although experimental H19 perturbation has been associated with changes in PD-L1 and CD155 expression and altered immune-cell cytotoxicity in HCC models, these findings remain predominantly preclinical and do not establish H19 as a predictive biomarker of benefit from immune checkpoint blockade. In particular, direct clinical evidence demonstrating that H19 levels predict response, resistance, or survival benefit following checkpoint inhibition remains limited. Associations between H19 expression and immune-related phenotypes should therefore be distinguished from evidence of treatment-specific predictive value.

Finally, several proposed H19-mediated ceRNA mechanisms remain mechanistically incompletely tested. Functional competition between H19, miRNAs, and downstream targets depends not only on molecular interaction or binding but also on the relative abundance of the competing RNAs, target-site availability, and miRNA–target stoichiometry. Quantitative studies of ceRNA biology have demonstrated that these parameters can critically determine whether competition is biologically meaningful [131]. Accordingly, reported H19–miRNA–target interactions should not automatically be interpreted as evidence of functional ceRNA regulation unless the relevant abundance and stoichiometric relationships have been experimentally evaluated.

These limitations indicate that the current evidence should be interpreted according to the level of experimental support available for each proposed relationship. In particular, direct mechanistic findings should be distinguished from pathways reconstructed from separate studies, observations derived predominantly from cell-line or preclinical models, associations reported in heterogeneous clinical samples, and mechanisms inferred from secondary literature. More rigorous validation in well-characterized in vivo and clinical systems, together with quantitative testing of ceRNA relationships and comprehensive characterization of immune-cell states, will be necessary to determine which H19-associated pathways are reproducible, context specific, and clinically actionable.

## Future direction

Although other lncRNAs, including Metastasis-Associated Lung Adenocarcinoma Transcript 1(MALAT1), CCAT1, XIST, and Nuclear Paraspeckle Assembly Transcript 1(NEAT1), have also been implicated in tumor progression and immune regulation, several features support the particular relevance of H19 to HCC and CRC [132]. H19 is an imprinted oncofetal transcript that is highly expressed during embryonic development, largely downregulated after birth, and aberrantly reactivated in several malignancies, providing a distinctive biological basis for its investigation as a tumor-associated molecule [133]. Moreover, H19 has been detected in circulating and extracellular vesicle-associated compartments and has been implicated in intercellular communication, tumor progression, metastasis, and treatment resistance in HCC and CRC [134]. Its relevance to HCC and CRC is further supported by its reported involvement in CRC liver metastasis and by the availability of an H19 promoter-directed therapeutic strategy, BC-819 [67, 135]. Importantly, BC-819 exploits H19 promoter activity to drive expression of a cytotoxic gene product rather than directly suppressing H19 RNA function; therefore, it should be distinguished conceptually from RNA-directed approaches such as antisense oligonucleotides, siRNAs, or CRISPR interference [136, 137].These features provide a rationale for focusing on H19 while recognizing that it functions within a broader network of immune-regulatory lncRNAs [132]. Indeed, H19, CCAT1, and MALAT1 are concurrently upregulated in the sera, PBMCs, and tissues of patients with HCC, emphasizing the need for future comparative studies to determine whether H19 has distinct or overlapping functions relative to other immune-associated lncRNAs [80]. Given tumor plasticity and the complexity of immune regulation, resistance to H19-targeted strategies could potentially emerge through compensatory activity of other lncRNAs, including XIST and MALAT1, or through alternative pathways regulating PD-L1 [132]. Future studies should longitudinally assess H19, PD-L1, immune profiles, and related non-coding RNA networks to identify potential resistance mechanisms. With sufficient preclinical validation, H19-targeted strategies could then be evaluated in biomarker-selected HCC and CRC patients to determine their potential role in precision immuno-oncology.

Beyond these mechanistic gaps, two fundamental questions remain unresolved. First, does H19 directly cause PD-L1 upregulation in HCC and CRC, or do their expression patterns reflect indirect or parallel regulatory mechanisms? Second, are the proposed miRNA axes involving miR-200a, miR-140-5p, miR-34a, and miR-29b-3p functionally redundant, or does each exert distinct effects depending on the tumor and tissue context? Conditional H19 knockout in syngeneic CRC models, including Azoxymethane/Dextran Sodium Sulfate (AOM/DSS)-induced tumors, and HCC models, such as Diethylnitrosamine (DEN)-induced tumors, combined with H19 rescue and miRNA-mimic rescue experiments, could help establish causality and clarify tissue-specific pathway dominance. For RNA-directed approaches, specificity should also be demonstrated using independent H19-targeting sequences, appropriate scrambled controls, and rescue experiments in which H19 expression or function is restored; reversal of the antitumor phenotype after H19 restoration would provide stronger evidence that the therapeutic effect is attributable to H19 suppression rather than nonspecific oligonucleotide activity. Such studies should also directly assess changes in PD-L1 expression, immune-cell composition, and immune-cell function, together with responses to PD-1/PD-L1 blockade, to determine whether H19 inhibition can meaningfully alter antitumor immunity. Importantly, combination studies should determine whether H19 inhibition provides additional benefit beyond its direct effects on tumor-cell proliferation, for example by demonstrating changes in immune-cell recruitment, activation, cytotoxicity, or exhaustion that are associated with enhanced checkpoint blockade efficacy.

Future therapeutic development should distinguish among promoter-directed strategies that suppress H19-driven transcription, RNA-directed approaches that directly reduce H19 RNA abundance or function, and delivery strategies designed to achieve tumor-selective exposure of H19-targeting agents. Even if H19 is validated as a functional regulator of immune escape, tumor-specific delivery remains a major obstacle to therapeutic translation. Conventional antisense oligonucleotides and small interfering RNAs(siRNAs) may face limitations related to stability, cellular uptake, biodistribution, and off-target exposure [138]. Extracellular vesicles (EVs), which naturally mediate intercellular communication and can transport regulatory RNAs, represent one potential delivery platform, although their clinical application remains technically challenging [139]. Surface engineering has shown preclinical promise; for example, Human Embryonic Kidney 293 Cells (HEK293)-derived EVs displaying Glypican-3(GPC3)-targeting nanobodies enhanced tumor specificity and reduced hepatic accumulation in preclinical HCC models [140]. For targeting tumor-associated macrophages, CD206-binding peptides have been used to engineer exosomes for diagnostic and therapeutic applications and to facilitate mRNA delivery to M2-polarized macrophages in vivo [141]. CRC-derived exosomes functionalized with A33 antibodies have also been investigated for tumor detection, although their application as in vivo therapeutic carriers for anti-H19 cargo have not been established [142].

H19-targeted therapy remains largely exploratory. BC-819 (DTA-H19), a plasmid construct that exploits the H19 promoter to drive expression of the diphtheria toxin A chain in H19-positive tumor cells, has been evaluated in phase I/IIa and IIb clinical trials for bladder cancer, demonstrating complete and partial response rates of 22% and 44%, respectively. Preclinical studies have also reported antitumor activity in pancreatic cancer and colorectal cancer liver metastases [135]. However, these early-phase findings were obtained primarily in bladder cancer and demonstrate the feasibility and preliminary activity of an H19 promoter-directed cytotoxic strategy rather than clinical validation of H19 RNA as a therapeutic target. They cannot be directly extrapolated to HCC or CRC, nor do they establish that direct suppression of H19 RNA would produce similar antitumor effects. Consequently, despite encouraging early-phase findings, the therapeutic potential of BC-819 remains invalidated in large, randomized clinical studies. Furthermore, BC-819 selectively eliminates H19-expressing tumor cells through H19 promoter-driven toxin expression rather than directly inhibiting H19 RNA function. Importantly, BC-819 does not provide evidence that direct H19 RNA suppression can reverse PD-L1-mediated immune suppression, remodel the tumor immune microenvironment, or sensitize tumors to immune checkpoint blockade [142]. Whether direct H19 RNA inhibition can enhance responses to PD-1/PD-L1 blockade therefore remains an untested therapeutic hypothesis requiring dedicated preclinical validation.

## Conclusion

H19 has attracted increasing attention as a potential interface between long non-coding RNA-mediated regulation, tumor-intrinsic signaling, and immune remodeling in hepatocellular carcinoma (HCC) and colorectal cancer (CRC). Available evidence indicates that H19 can influence tumor-associated phenotypes through diverse mechanisms, including ceRNA-associated interactions, RNA–protein regulation, extracellular-vesicle-mediated communication, and oncogenic signaling pathways. However, the extent to which these mechanisms converge directly on PD-1/PD-L1 signaling remains incompletely defined. Although selected experimental studies provide evidence that H19 perturbation can affect PD-L1-associated phenotypes, a complete, mechanistically resolved H19-dependent PD-L1 regulatory axis has not been established in HCC or CRC, and several proposed connections remain indirectly supported, contextual, or hypothetical.

Important uncertainties therefore remain regarding the causal relationships among H19, immune checkpoint regulation, and tumor microenvironmental remodeling, as well as their clinical relevance. The established biology of PD-1/PD-L1 signaling should be distinguished from the more limited evidence linking H19 specifically to T-cell dysfunction, macrophage remodeling, or other immune-cell states. In HCC, the context-dependent behavior of H19 warrants particular consideration, as both tumor-promoting and tumor-suppressive effects have been reported depending on biological and disease context. In CRC, H19 has frequently been associated with oncogenic phenotypes, but its significance may likewise vary according to molecular subtype, treatment setting, metastatic context, and cellular compartment. Clinical associations involving circulating H19 remain preliminary, and H19 has not yet been clinically validated as a predictive biomarker of response to immune checkpoint blockade.

Accordingly, H19 should be regarded as a biologically plausible and experimentally testable candidate interface within tumor–immune regulatory networks rather than as an established regulator of PD-1/PD-L1 signaling. The principal value of this review is therefore not to establish a single H19–PD-L1 pathway, but to provide a critical evidence map distinguishing experimentally demonstrated findings from indirect, contextual, and hypothetical relationships. This framework highlights the need for disease-specific mechanistic studies incorporating H19 perturbation, quantitative PD-L1 assessment, pathway-specific rescue or epistasis, and immune-functional validation, together with spatial and single-cell approaches and prospective clinical studies to determine whether H19 can ultimately have predictive or therapeutic relevance for immune checkpoint blockade.

## Data Availability

No datasets were generated or analysed during the current study.

## Declarations

### AI declaration

ChatGPT and Grammarly were used to paraphrase and summarize sentences.
